# A Potential Use of Vidarabine: Alleviation of Functional Constipation Through Modulation of the Adenosine A2A Receptor-MLC Signaling Pathway and the Gut Microbiota

**DOI:** 10.3390/ijms252312810

**Published:** 2024-11-28

**Authors:** Xiaoyu Gao, Kaifeng Guo, Shuangfeng Liu, Weixing Yang, Jun Sheng, Yang Tian, Lei Peng, Yan Zhao

**Affiliations:** 1 Yunnan Key Laboratory of Precision Nutrition and Personalized Food Manufacturing, Yunnan Agricultural University, Kunming 650201, China; 2018014@ynau.edu.cn (X.G.); tianyang@ynau.edu.cn (Y.T.); 2 College of Food Science and Technology, Yunnan Agricultural University, Kunming 650201, China; guo0446@163.com (K.G.); 2021210106@stu.ynau.edu.cn (S.L.); 2021110006@stu.ynau.edu.cn (W.Y.); 3 Engineering Research Center of Development and Utilization of Food and Drug Homologous Resources, Ministry of Education, Yunnan Agricultural University, Kunming 650201, China; shengj@ynau.edu.cn; 4 Division of Science and Technology, Yunnan Agricultural University, Kunming 650201, China

**Keywords:** intestinal motility, gut microbiota, Muribaculaceae, *Muribaculum*, *Parasutterella*, adenine arabinoside, aquaporins, adenosine A2A receptor

## Abstract

Vidarabine (VID) is an antiviral medication that is commonly utilized to treat conditions such as hand, foot, and mouth disease and herpes. Constipation is a prevalent complication of these diseases. Could VID treat these diseases by influencing defecation behavior? To date, no studies have been conducted on the potential of VID to relieve constipation. Therefore, a systematic investigation was conducted into the laxative effects and mechanisms of VID using loperamide-induced functional constipated mice. The findings indicate that the oral administration of VID promoted gastrointestinal peristalsis, improved fecal properties, facilitated defecation, and demonstrated a significant laxative effect on functional constipated mice. It has been demonstrated that VID may increase the water content of feces by regulating the expression of aquaporins (AQP3, AQP4, and *AQP8*) in the colon and promote intestinal motility by regulating the expression of neurotransmitters (AChE and VIP) and the adenosine A2A receptor–myosin light chain (A2AR-MLC) signaling pathway in constipated mice. Concurrently, VID may also reduce colonic inflammation in constipated mice, reinforce the gut barrier function, and alter the composition and structure of the gut microbial community. Some microbial taxa, including Firmicutes and *Lactobacillus*, were found to be associated with the alleviation of constipation, while other taxa, including Bacteroidetes, Proteobacteria, Muribaculaceae, *Muribaculum*, *norank__f__Desulfovibrionaceae*, and *Parasutterella*, were found to be associated with constipation. These results indicate that the gut microbiota may play a significant role in the alleviation of constipation by VID. These findings confirm the efficacy of VID in a constipated animal model, which justifies further investigation into its potential clinical applications.

## 1. Introduction

Functional constipation (FC), one of the most prevalent forms of constipation and a complication of numerous diseases, significantly affects the quality of life of patients. Despite extensive research on the pathogenesis of functional constipation in modern medicine, the underlying mechanisms remain poorly understood. In addition to dietary patterns and lifestyle habits, the enteric nervous system (ENS), smooth muscle dysfunction, gut microbiota, and intestinal water–fluid balance have all been implicated in the pathogenesis of FC [1,2,3,4]. The regulation of gastrointestinal motility is mainly dependent on the ENS.

The ENS can regulate intestinal motility through the secretion of excitatory neurotransmitters and inhibitory neurotransmitters. Additionally, the ENS interacts with the immune system, intestinal secretions, gut microbiota, and fermentation products, which in turn regulate intestinal motility [5]. Smooth muscle dysfunction is also an important causative factor in FC. Fecal transit through the intestinal lumen is dependent on the movement of the intestinal muscles. Consequently, many disorders that directly affect intestinal smooth muscle function lead to constipation [6,7].

The balance of water metabolism in the intestine is also closely related to FC. The aquaporins are a class of transmembrane protein in intestinal epithelial cells that function as a “water pump” in the intestinal tract, regulating the in-and-out movement of water and maintaining the water–liquid balance. This process is essential for regulating intestinal water metabolism [8,9]. A growing body of evidence supports the critical role of the gut microbiota in regulating gut motility [10,11]. Previous studies have demonstrated that bacterial colonization of the gut is critical for the development and maturation of the ENS [12], and that an abnormal composition of the gastrointestinal microbiota may lead to disruption in “microbe–gut–brain axis” signaling, resulting in altered gut motility [13,14].

Vidarabine (adenine arabinoside, VID), the chemical compound 9-β-D-arabinofuranosyladenine, is a drug with documented antiviral activity. In addition, VID has been shown to possess a range of other biological effects, including antitumor, immunomodulatory, and cardioprotective properties [15,16,17]. Scientists have demonstrated, through acute and subacute toxicity tests, that VID has no adverse effects on major organs such as the heart, liver, and kidneys [18]. Therefore, VID is still employed in countries such as China and Japan. Adenosine and its analogs can act in vivo through adenosine receptors, which are G protein-coupled receptors. These receptors are categorized into four subtypes: A1R, A2AR, A2BR, and A3R [19,20]. Among these, A2A receptors are predominantly expressed at the terminals of cholinergic nerves and play a pivotal role in maintaining cholinergic neurotransmission and regulating gastrointestinal motility [21]. As an analog of adenosine, VID may also be able to modulate the intestinal motility by acting on the A2A receptors, but no studies have been reported to date in this regard.

VID is a commonly used treatment for a number of diseases, including hand, foot, and mouth disease, herpes, herpes simplex virus encephalitis, and hepatitis B. These diseases frequently present with constipation [22,23,24]. However, it remains uncertain whether VID can influence intestinal motility and bowel behavior beyond its established antiviral effects. To date, no studies have been conducted on the effects of VID on constipation. To this end, the present study sought to evaluate the laxative effect of VID using a mouse model of loperamide-induced functional constipation and to elucidate its potential laxative mechanism given various aspects, including the ENS, intestinal water and fluid metabolism, intestinal inflammation, gut barrier, and gut microbiota. The results may provide a theoretical foundation for the application of VID as a potential laxative drug in specific disease populations.

## 2. Results

### 2.1. VID Attenuates Symptoms of Loperamide-Induced Constipation

**Figure 1:**
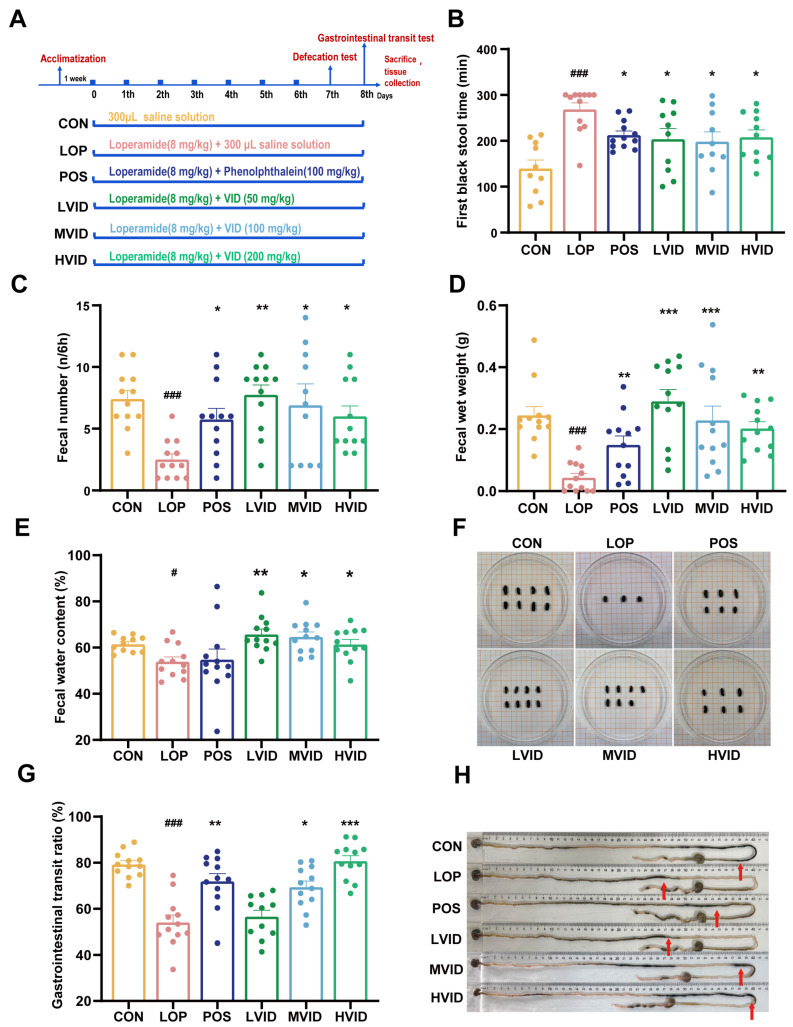
VID alleviates loperamide-induced constipation in mice. (**A**) Basic workflow and grouping of the animal experiment. (**B**) The first black stool time (FBST). (**C**) Fecal number (FN). (**D**) Fecal wet weight (FW). (**E**) Fecal water content (FWC). (**F**) Representative morphological characterization of the feces in each group. (**G**) Gastrointestinal transit rate (GTR). (**H**) Representative pictures of ink advance in each group, red arrows point to where the ink reaches. The data are presented as the mean ± SEM (*n* = 12). #, vs. CON group; *, vs. LOP group. #, *p* < 0.05; ###, *p* < 0.001. *, *p* < 0.05; **, *p* < 0.01; ***, *p* < 0.001.

The course of the animal experiment is shown in Figure 1A. Compared with the CON group, mice in the LOP group showed a significant increase in FBST (Figure 1B) and a significant decrease in FW and FN within 6 h (Figure 1C,D). Meanwhile, loperamide also caused a significant decrease in FWC (Figure 1E), and VID treatment significantly shortened FBST (Figure 1B), increased FN (Figure 1C), and reversed the morphological characterization of feces in constipated mice (Figure 1D–F). In the gastrointestinal transit experiment, loperamide resulted in a significant decrease in the gastrointestinal transit rate (GTR) of mice, whereas GTR increased in a dose-dependent manner after VID intervention, and the effect of medium- and high-dose VID treatments in promoting small intestinal motility was significant (Figure 1G,H, *p* < 0.05).

During the experimental period, all groups of mice exhibited weight gain. However, the mice in the LOP group exhibited the least weight gain (Figure S1A), and the loperamide intervention affected the intake and water consumption of the mice (Figure S1B,C). VID did not significantly affect liver or kidney indices or the length of the small intestine (Figure S1D–F), which indicates that none of the subjects significantly affected the basal organismal indices of the mice. In conclusion, the results demonstrate that VID significantly alleviated the symptoms of loperamide-induced constipation.

### 2.2. VID Regulates the Expression of Aquaporins in the Colon

**Figure 2:**
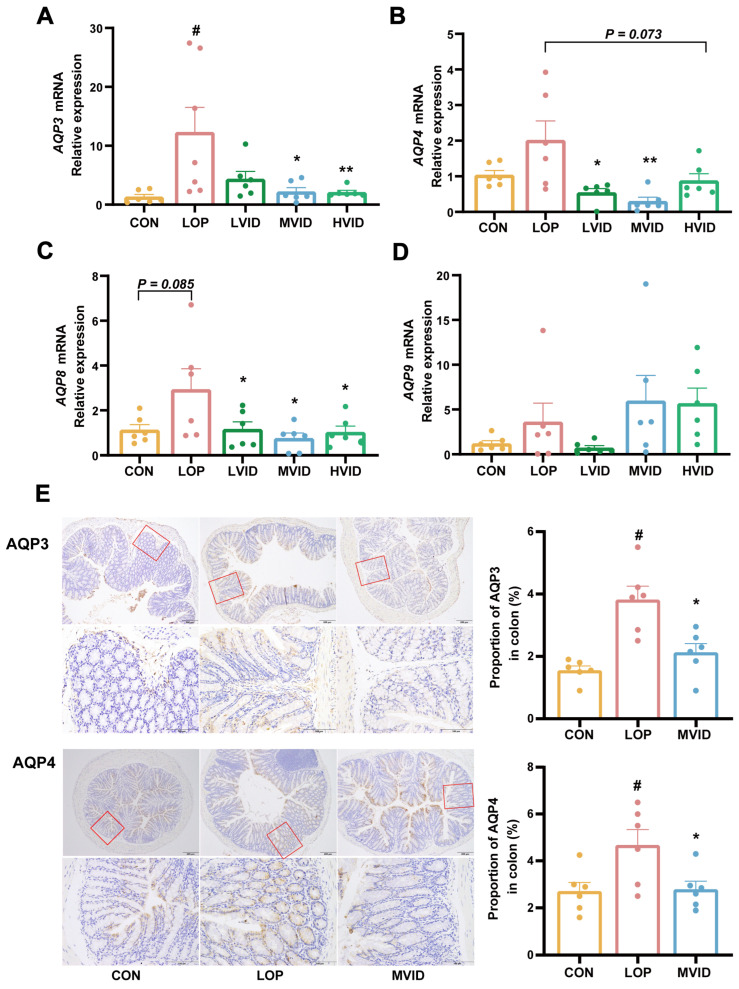
Effect of VID on the expression of aquaporins in the colons of mice with constipation. (**A**) The relative expression of *AQP3*. (**B**) The relative expression of *AQP4*. (**C**) The relative expression of *AQP8*. (**D**) The relative expression of *AQP9*. (**E**) The immunohistochemical analysis of colonic AQP3 and AQP4 in mice. The data are presented as the mean ± SEM (*n* = 6). #, vs. CON group; *, vs. LOP group. #, *p* < 0.05. *, *p* < 0.05; **, *p* < 0.01.

Aquaporins are closely related to the water–fluid metabolism in the intestine. Animal experiments have demonstrated that VID can enhance the fecal water content in constipated mice (Figure 1E). RT-qPCR results demonstrate that loperamide significantly upregulated the mRNA expression of *Aquaporin 3* (*AQP3*) (*p* < 0.05, Figure 2A) and also enhanced the expression of *Aquaporin 4* (*AQP4*), *Aquaporin 8* (*AQP8*), and *Aquaporin 9* (*AQP9*, Figure 2B–D). The administration of different doses of VID was found to inhibit the mRNA expression of *AQP3*, *AQP4*, and *AQP8* in the colons of constipated mice to varying degrees. The inhibitory effect of the medium dose of VID (MVID group) on these *AQPs* was found to be most stable (Figure 2A–C). The immunohistochemical results demonstrate that medium-dose VID markedly suppressed the protein expression of AQP3 and AQP4 in the colons of mice with constipation.

### 2.3. VID Modulates the Adenosine A2A Receptor Signaling Pathway and Neurotransmitter Expression

**Figure 3:**
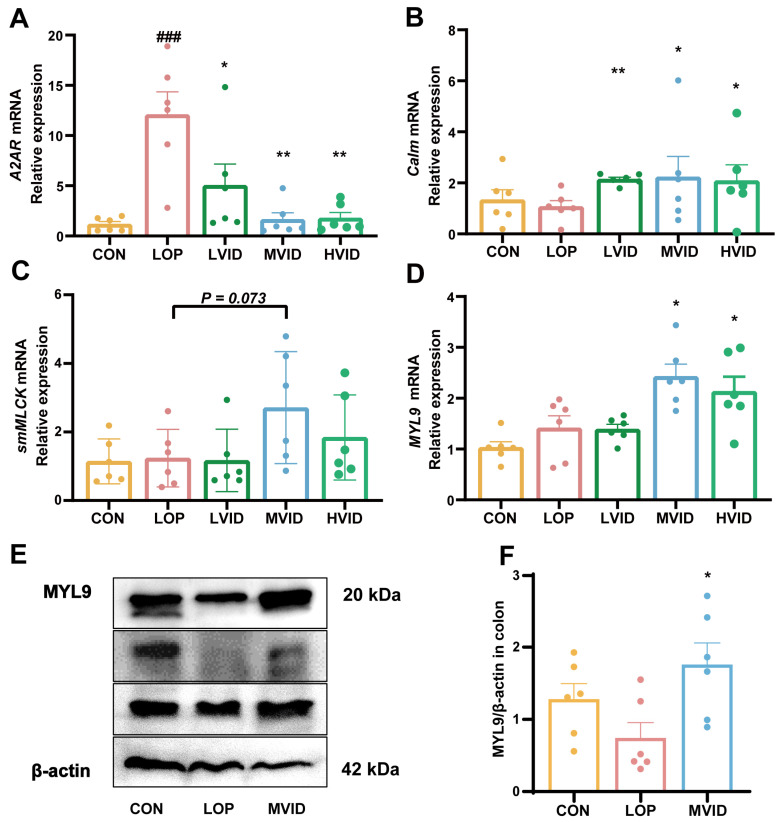
VID reduces A2AR expression and enhances CALM-MLCK-MLC signaling pathway. (**A**) The relative expression of *A2AR*. (**B**) The relative expression of *Calm*. (**C**) The relative expression of *smMLCK*. (**D**) The relative expression of *MYL9*. (**E**,**F**) The relative protein expression of MYL9 in the colon. The data are presented as the mean ± SEM (*n* = 6). #, vs. CON group; *, vs. LOP group. ###, *p* < 0.001. *, *p* < 0.05; **, *p* < 0.01.

It has been demonstrated that adenosine can modulate downstream signaling pathways to inhibit intestinal motility by binding to the A2A receptor (A2AR) [25,26]. MYL9, a regulatory light chain of myosin, is a pivotal link in the activation of the motility of myosin, which can provide a driving force for cell motility. VID is an isomer of adenosine. We found that loperamide significantly upregulated the mRNA expression of the A2A receptor in mouse colonic tissues, whereas VID significantly inhibited the mRNA expression of the A2AR in a dose-dependent manner (*p* < 0.05, Figure 3A).

Meanwhile, we observed that low, medium, and high doses of VID significantly upregulated the mRNA expression of *calmodulin* (*Calm*) (*p* < 0.05, Figure 3B). The medium-dose VID also upregulated the gene expression of smooth muscle myosin light chain kinase (*smMLCK*, *p* = 0.077, Figure 3C), and medium- and high-dose VID significantly upregulated the expression of *Myosin light chain 9* (MYL9, *p* < 0.05, Figure 3D). Consistently, VID was similarly able to significantly upregulate the protein expression level of MYL9 (*p* < 0.05), completely reversing the inhibitory effect of loperamide (Figure 3E,F). These findings suggest that VID promotes intestinal motility by modulating the A2AR-CALM-MLCK-MLC signaling pathway.

In addition, we examined the expression of neurotransmitter-related factors in the serum and colons of mice. The medium dose of VID significantly reduced the serum levels of acetylcholinesterase (AChE) and vasoactive intestinal peptide (VIP) in constipated mice (Figure S2A,B), as well as the mRNA expression of *AChE* and *VIP* in the colon (Figure S2C,D). This suggests that VID may promote intestinal motility and alleviate constipation by increasing the expression of excitatory neurotransmitters and decreasing the expression of inhibitory neurotransmitters.

### 2.4. VID Reduces Colonic Inflammation and Enhances the Gut Barrier Function

**Figure 4:**
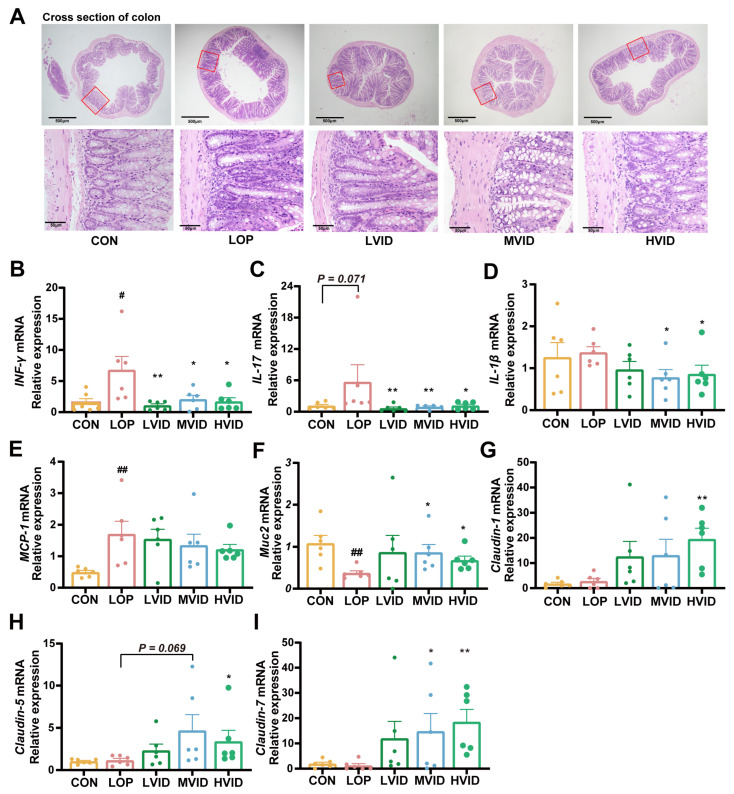
Effect of VID on the gene expression of inflammatory and barrier factors in the colons of constipated mice. (**A**) H&E staining of colonic tissues; (**B**–**E**) The relative mRNA expression of inflammatory factors *INF-γ*, *IL-17*, *IL-1β*, and *MCP-1*, respectively; (**F**–**I**) The relative mRNA expression of gut barrier factors *Muc 2*, *Claudin-1*, *Claudin-5*, and *Claudin-7*. The data are presented as the mean ± SEM (*n* = 6). #, vs. CON group; *, vs. LOP group. #, *p* < 0.05; ##, *p* < 0.01. *, *p* < 0.05; **, *p* < 0.01.

From the pathological sections of the colon, it could be observed that there was inflammatory cell infiltration in the colon tissue of loperamide-induced constipated mice. In addition, the villi of the colon tissue of the mice in the VID intervention group were arranged in an orderly manner, and inflammatory cell infiltration was alleviated (Figure 4A). The results of the RT-qPCR assay show that loperamide induced an increase in the relative expression of *INF-γ*, *IL-17*, and *MCP-1* mRNA in mouse colon tissues, whereas VID treatment downregulated the relative expression of *INF-γ*, *IL-17*, and *IL-1β* mRNA in the colons of constipated mice. The downregulation effect was most significant with medium- and high-dose treatments (*p* < 0.05, Figure 4B–E).

Inflammation causes intestinal barrier damage, and intestinal barrier damage exacerbates intestinal inflammation. Loperamide also significantly inhibited the mRNA expression of the barrier factor Mucin 2 (*Muc2*) in mouse colons but had less of an effect on *Claudin-1*, *Claudin-5*, and *Claudin-7*. VID treatment upregulated the expression of these barrier factors, and again, the upregulation effect was most pronounced with the medium- and high-dose treatments (Figure 4F–I). These results suggest that VID attenuated loperamide-induced colonic inflammation and improved intestinal barrier function.

### 2.5. VID Remodels the Gut Microbiota in FC Mice

**Figure 5:**
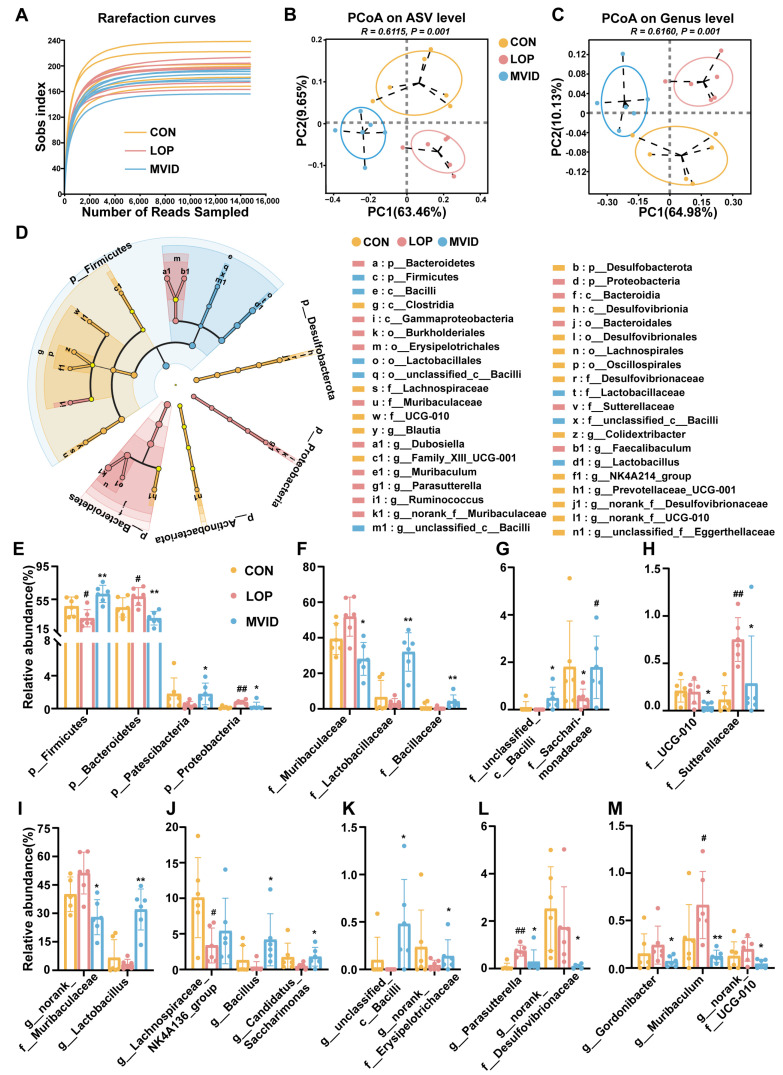
VID alters the structure and composition of gut microbial communities in FC mice. (**A**) Rank–abundance curves; (**B**,**C**) PCoA plot at the ASV level and genus level based on the weighted Unifrac algorithm; (**D**) LEfSe analysis (phylum to genera, LDA score > 2.0); (**E**–**M**) analysis of differences between groups at the phylum, family, and genus level. The data are presented as the mean ± SEM, *n* = 6. #, vs. CON group; *, vs. LOP group. #, *p* < 0.05; ##, *p* < 0.05. *, *p* < 0.05; **, *p* < 0.01.

High-throughput sequencing of the 16Sr RNA gene was used to evaluate the effects of loperamide and VID on the gut microbiota. As illustrated in Figure 5A, the Sobs rarefaction curve exhibited a plateau when the number of sample reads reached 10,000, indicating that the results of this sequencing were reliable and could reflect the vast majority of bacterial diversity information in the samples. The impact of loperamide and VID on α-diversity was minimal (Figure S3), whereas their influence on β-diversity was pronounced. The samples from the CON, LOP, and VID groups exhibited enhanced separation in PCoA plots (Figure 5B,C, *p* < 0.05), suggesting that LOP and VID treatments exerted a notable impact on the structure and composition of the gut microbiota. This hypothesis was corroborated by the results of the LEfSe analysis and microbial composition analysis (Figure 5D and Figure S4). The three groups of CON, LOP, and VID had their own dominant microbial taxa, with a total of 40 species from phylum to genus (Figure 5D and Figure S5).

This study focused on microbial taxa that exhibited significant alterations in response to VID. At the phylum level, VID resulted in a notable reduction in the relative abundance of Bacteroidetes and Proteobacteria, accompanied by an increase in the relative abundance of Firmicutes and Patescibacteria in the cecum of loperamide-induced constipated mice (*p* < 0.05, Figure 5E). At the family level, VID significantly increased the relative abundance of dominant microbial taxa, including Lactobacillaceae, Bacillaceae, f_unclassified_c__Bacill, and Saccharomycetaceae, in the cecum of constipated mice (*p* < 0.05). Figure 5E–H illustrates that VID reduced the relative abundance of dominant taxa, such as Muribaculaceae, UCG-010, and Sutterellaceae (*p* < 0.05).

At the genus level, *Lactobacillus*, *Bacillus*, *Candidatus_Saccharimonas*, *g__unclassified__c__Bacilli*, and *g__norank__f__Erysipelotrichaceae* exhibited a significant upward shift in relative abundance in response to VID (*p* < 0.05, Figure 5I–K). In contrast, g*__norank__f__Muribaculaceae*, *Parasutterella*, *g__norank__f__Desulfovibrionaceae*, *Gordonibacter*, *Muribaculum*, and *g__ norank__f__UCG-010* exhibited a significant downward shift in relative abundance (*p* < 0.05, Figure 5I,L,M). These results indicate that VID alters the structure of the cecum microbial community in loperamide-induced constipated mice by influencing the relative abundance of specific microbial taxa.

### 2.6. Correlation Analysis of Differential Microbial Taxa with Host Parameters

Bivariate correlation analysis (Spearman) was used to construct correlations between differential microbial taxa and phenotypic indicators of constipation, gastrointestinal motility factors, aquaporins, and gut barrier factors.

**Figure 6:**
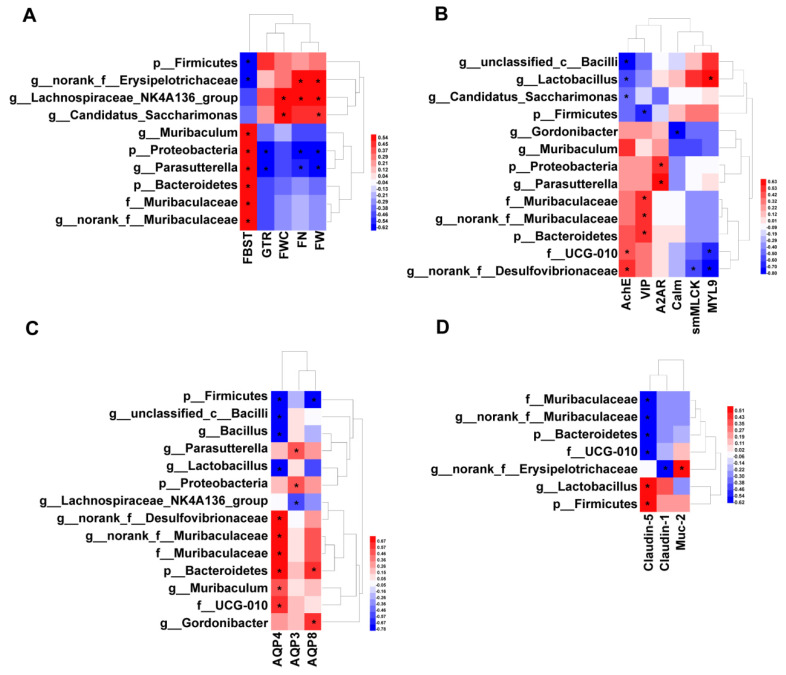
Heatmaps showing correlations between the differential microbial taxa and main host parameters. (**A**) Correlations between the specific taxa and the phenotypic indicators of constipation, including FBST, FN, FW, FWC, and GTR. (**B**) Correlations between the specific taxa and the A2AR-MLC signaling pathway and neurotransmitter expression. (**C**) Correlations between the specific taxa and *AQPs*. (**D**) Correlations between the specific taxa and the gut barrier factors. The color at each point of intersection indicates the value of the *r* coefficient. *Benjamini*–*Hochberg* (BH) procedures were used to adjust *p*-values for multiple testing; * indicates a significant correlation between these two parameters (*p* < 0.05).

As shown in Figure 6A, Firmicutes and its inclusion *g__norank__f__Desulfovibrionaceae* were significantly negatively correlated with FBST, whereas Bacteroidetes and its inclusions Muribaculaceae, *g__norank__f__Muribaculaceae*, and *Muribaculum* were significantly positively correlated with FBST. Proteobacteria and its inclusion *Parasutterella* were also significantly positively correlated with FBST, but significantly negatively correlated with important phenotypic indicators of constipation such as GTR, FN, and FW.

Additionally, several microbial taxa were found to be associated with the expression of neurotransmitters and colonic adenosine A2A receptor-associated gastrointestinal motility factors (Figure 6B). *Lactobacillus* was significantly negatively correlated with AChE and significantly positively correlated with MYL9, whereas UCG-010 and *g__norank__f__Desulfovibrionaceae* exhibited the opposite patterns. Proteobacteria and their inclusion *Parasutterella* were significantly positively correlated with A2AR. Bacteroidetes and its inclusions, Muribaculaceae and *g__norank__f__Muribaculaceae*, exhibited a significant positive correlation with VIP, whereas Firmicutes exhibited a significant negative correlation with VIP.

The correlation between VID-shaped differential microbial taxa and *AQPs* expression exhibited two principal forms (Figure 6C). The Firmicutes and their inclusions of *Lactobacillus*, *Bacillus*, and *g__unclassified__c__Bacilli* were significantly negatively correlated with *AQP3*, while the Bacteroidetes and its inclusions of Muribaculaceae, *g__norank__f__Muribaculaceae*, and *Muribaculum* were significantly positively correlated with *AQP3* (Figure 6C).

It is noteworthy that the results of the correlation analysis of Firmicutes and Bacteroidetes with gut barrier factors exhibit a similar pattern to that observed in the host indicators described above (Figure 6D). The dominant microbial taxon, *Lactobacillus*, was significantly positively correlated with *Claudin-5*, while Muribaculaceae was significantly negatively correlated with *Claudin-5* (Figure 6D). In summary, we hypothesize that a number of microbial taxa might play important roles in VID’s regulation of defecation behavior, perhaps with division of labor and cooperation.

## 3. Discussion

Loperamide is a commonly used pharmaceutical agent in clinical settings for the treatment of diarrhea. It has been demonstrated to significantly reduce gastrointestinal transmission and bowel frequency [27]. Loperamide has been shown to reduce fecal water content and fecal pellet numbers, decrease the gastrointestinal transit rate, and provoke symptoms similar to those of constipation in clinical patients [28]. The mouse constipation model constructed with loperamide in this study exhibited consistent clinical symptoms of functional constipation and did not result in adverse phenomena such as the death of mice during the experimental process, indicating that the constipation mouse model was successfully established. In the present work, we explored the effects of VID on major constipation phenotypic indices, such as FBST, FN, FW, FWC, and GTR, mainly through defecation experiments and gastrointestinal transit experiments. The results demonstrate that VID significantly reduced the FBST, increased the FWC, and enhanced gastrointestinal motility in constipated mice. These findings provide direct evidence of the laxative effect of VID.

The notable increase in fecal water content is indicative of the laxative properties of VID. This suggests that VID may be effective at alleviating constipation through regulating the water metabolism in the intestinal lumen. The AQPs, a class of cell membrane channel proteins that can efficiently and selectively transport water molecules, are widely distributed in the epithelial cells of gastrointestinal tract tissues in humans and other mammals [29]. These proteins regulate intestinal water metabolism while also secreting mucus to protect and lubricate the intestinal tract [30]. To date, a total of 13 AQPs have been identified in mammals [31]. Of these, the most closely related to constipation are AQP3, 4, 8, and 9.

AQP3 is expressed primarily in intestinal mucosal epithelial cells, which facilitate the transport of water from the intestinal lumen to the cellular space, accelerate water translocation, and are directly linked to the development of constipation. Upregulation of AQP3 expression in colon epithelial cells can result in severe constipation [32]. Rhodopsin regulates water transport and absorption through the cAMP-dependent PKA/p-CREB signaling pathway, and alters the expression of AQP3, thereby alleviating the symptoms of constipation [33]. AQP4 is mainly located in the basement membrane of crypt cells and epithelial cells [34], and its elevated expression accelerates water absorption in the colonic mucosa. Wang et al. demonstrated that the knockout of the *AQP4* gene in mice resulted in a decrease in water absorption in the colon and an increase in water content in the feces, further supporting the hypothesis that AQP4 plays a role in water absorption in the colon [35].

AQP8 is expressed at high levels in the absorptive epithelial cells of the proximal jejunum, proximal colon, and rectum. It is confined to the cytoplasm beneath the parietal membrane and is not found on the basement membrane [32]. AQP9 is expressed on the basement membrane of mucosal cupping cells [36], which promotes mucus secretion, protects the colonic mucosa, and lubricates the bowel. The aberrant expression of AQPs in colonic epithelial cells affects the water content of feces and is a significant contributing factor in the pathogenesis of constipation [37,38,39]. Our results show that VID significantly increased the water content of feces in constipated mice while also significantly decreasing the expression of AQP3, AQP4, and *AQP8*. These results suggest that adenosine may regulate intestinal water metabolism, increase fecal water content, and alleviate constipation by affecting the expression of AQPs.

A2AR is a classic G protein-coupled receptor with a high affinity for adenosine. Extensive research has been conducted on the role of A2AR in cardiovascular function, inflammation, sleep/wake behavior, cognition, and other primary neurological functions [39]. Elevated A2AR appears to play a deleterious role in a number of disease states: it promotes neuroinflammation and astrocyte responses that can lead to the worsening of neurodegenerative and psychiatric disorders [40].

A2AR antagonists have been demonstrated to exert a significant influence on the control and reversal of synaptic dysfunction [41]. A2AR antagonists also result in vascular smooth muscle contraction [42]. A2AR coupled to Gs protein activates AC, leading to elevated intracellular cAMP levels and subsequent activation of Protein Kinase A (PKA) [26]. PKA activation can additionally inhibit intestinal motility through the Calm-MLC signaling pathway. Antonioli et al. proposed that A2AR modulates colonic excitatory cholinergic neural activity through facilitative control of the inhibitory nitrocellulinergic pathway, and that this modulation is enhanced during intestinal inflammation [25]. Adenosine can negatively regulate mouse duodenal motility by controlling neurotransmitter release via A1 or A2A receptors [43]. In this study, as an adenosine isomer, VID treatment significantly inhibited the high expression of *A2AR* in the colons of constipated mice, while upregulating the gene expression of *CALM*, *smMLCK*, and *MYL9*, as well as the protein expression level of *MYL9*, a key factor for smooth muscle motility control. This indicates that VID, in contrast to adenosine, may inhibit the expression of *A2AR*, which in turn alters the Calm-MLC signaling pathway and promotes intestinal motility.

It is worth mentioning that VID treatment also significantly reduced the serum levels of AChE and VIP as well as the mRNA expression of AChE and VIP in the colonic tissues of constipated mice. The effects of VID on these neurotransmitters may also be closely related to A2AR. This is because A2AR plays a key role in regulating functions, such as synaptic plasticity, cognition, motor activity, neuroinflammation, and cell death, and is also involved in the release of several neurotransmitters [44,45,46].

The development of constipation is often accompanied by intestinal inflammation and damage to the intestinal barrier. It has been demonstrated that patients with constipation exhibit increased intestinal permeability and elevated serum concentrations of ovalbumin [46]. Mice colonized with microbiota from constipated patients display aberrant defecation parameters and diminished Muc 2 expression and mucin release, indicating that constipation-induced ecological dysregulation leads to a compromised epithelial barrier [47]. The microbiota can directly regulate the expression of tight junction proteins, thereby influencing intestinal immune homeostasis [48].

An inflammatory state can affect gastrointestinal motility [49]. Researchers have demonstrated the inhibitory effect of IL-1β on intestinal motility [50,51]. Aubé et al. showed that a certain dose of IL-1β inhibited intestinal contractions in the jejunal and colonic strips of Wistar rats [50]. Short-term IL-1β treatment inhibited acetylcholine release from longitudinal intestinal smooth muscle in rats [51]. Additionally, several studies have shown a close relationship between IL-17 and intestinal motility: a reduction in IL-17 is associated with an increase in intestinal motility [52,53,54]. Similarly, INF-γ inhibits the contractile activity of intestinal smooth muscle cells. Some researchers have found that IFN-γ inhibits human intestinal smooth muscle cell contraction and response to cholinergic agonists [55]. Intestinal surgery has been shown to induce IFN-γ expression in the small intestines and colons of mice, and knockdown of IFN-γ has been demonstrated to attenuate the intestinal surgery-induced reduction in intestinal and colonic transit function [56]. Animal experiments have indicated that MCP-1 may also have an inhibitory effect on intestinal motility. In a rat model, inhibition of MCP-1 with neutralizing antibodies has been shown to partially attenuate the colonic contractions induced by carbachol-induced colitis [57]. In humans, higher levels of MCP-1 in patients undergoing colorectal surgery have been associated with prolonged time of recovery of bowel function [58]. In this study, VID treatment resulted in a downregulation of the relative mRNA expression of INF-γ, IL-17, and IL-1β in the colons of constipated mice, an upregulation of the expression of the barrier factors Muc 2, Claudin-1, Claudin-5, and Claudin-7, and a reduction in the infiltration of inflammatory cells in the colon. Consequently, it can be postulated that how VID alleviates FC is inextricably linked to its ameliorative effects on intestinal inflammation and the intestinal barrier function.

It is generally accepted that the composition of the gut microbiota of constipated patients is significantly different from that of normal subjects, and the species diversity of the microbiota in patient samples is lower than that of healthy subjects [59]. Firmicutes and Bacteroidetes are the major microbial taxa at the phylum level in most mammals, including humans and rodents. Consequently, a notable alteration in the proportion of Firmicutes and Bacteroidetes, or the Firmicutes-to-Bacteroidetes ratio (F/B ratio), signifies a comprehensive transformation in the composition of the entire gut microbial community.

Previous studies have observed significant changes in microbial taxa in both humans [11,60,61,62] and other animals with constipation [63,64,65,66]. These changes can be reversed by certain foods, drugs, or probiotics. For instance, glucan-rich snail mucin heteropolysaccharides have been shown to modulate loperamide-induced constipation by increasing Firmicutes and decreasing Bacteroidetes [63]. A proprietary Chinese medicine, Shouhui Tongbian capsule, has been demonstrated to increase the relative abundance of *Lactobacillus* and the F/B ratio [64]. Fu brick tea aqueous extracts have been demonstrated to alleviate constipation while increasing the F/B ratio and the relative abundance of *Lactobacillus* [65]. In addition, the bacterial community structure of the intestinal mucosa was altered in spleen-deficient constipated mice, characterized by a decrease in the F/B ratio and an enrichment of Proteobacteria [66]. An increased abundance of Proteobacteria and Bacteroidetes has also been observed in constipation associated with Parkinson’s disease [67].

In this study, VID significantly decreased the relative abundance of Bacteroidetes and Proteobacteria and increased the relative abundance of Firmicutes in the cecum of loperamide-induced constipated mice. These findings are consistent with those of previous studies, further supporting the hypothesis that these microbial taxa may play an important role in the alleviation of constipation by VID. Furthermore, a correlation analysis revealed significant correlations between Firmicutes, Bacteroidetes, and Proteobacteria and constipation phenotypes.

*Lactobacillus* is a member of the Firmicutes phylum and is a well-known group of probiotics that are often reduced in the gastrointestinal tract of constipated animals. Foods and medications can also often alleviate constipation symptoms by increasing the abundance of *Lactobacillus* [68]. The supplementation of a variety of *Lactobacillus*, including *L. plantarum*, *L. paracasei*, *L. reuteri*, and *L. rhamnosus*, has been demonstrated to be an effective means of relieving constipation in animals [69,70,71,72]. In the present study, VID was also found to significantly increase the relative abundance of *Lactobacillus*. Furthermore, we observed a significant correlation between the relative abundance of *Lactobacillus* and AChE levels in serum, as well as the mRNA expression of *MYL9* and *AQP3* in the colons of constipated mice. These findings are consistent with those of previous studies. For instance, *L. reuteri* 1 has been demonstrated to enhance enterotoxigenic *Escherichia coli* K88-induced intestinal epithelial barrier function and alleviate inflammatory responses through the inhibition of the MLCK signaling pathway [73]. *L. acidophilus* can affect intestinal smooth muscle contraction in traumatic brain-injured mice via the PKC/MLCK/MLC signaling pathway [74]. *L. plantarum* CQPC02 has been shown to regulate the expression of AChE and AQPs in constipated mice [75].

In this study, we observed a significant decrease in the relative abundance of a number of potentially pathogenic bacteria in response to VID treatment. These taxa include Muribaculaceae, *Muribaculum*, *g__norank_f__Desulfovibrionaceae*, and *Parasutterella*. Moreover, these taxa demonstrated strong correlations with constipation phenotypic indicators, gastrointestinal motility factors, *AQPs*, and gut barrier factors. Muribaculaceae is a dominant family of Bacteroidetes. Some results indicated that Dioscin restores gut microbial diversity and the microbial community structure and increases the abundance of Muribaculaceae while relieving slow-transmission constipation in mice [76]. Our previous study also revealed that Muribaculaceae may play an important role in the relief of FC caused by *Cymbopogon citratus* aqueous extract [77].

Previous findings suggest that *Muribaculum* might also be a microbial taxon that can cause constipation. Complement 3 knockout mice were spontaneously constipated and microecologically dysregulated, and *Muribaculum* was increased during this process [78]. Additionally, dietary inulin could downregulate the abundance of *Muribacalum* in alleviating constipation-induced depression and anxiety behaviors [79].

*Parasutterella* is a significant member of the Proteobacteria and was previously believed to be associated with early constipation. However, following the consumption of red papaya, the relative abundance of *Parasutterella* returned to a healthy level [80]. *Cannabis sativa* L. aqueous extracts have been demonstrated to alleviate loperamide-induced constipation in mice by modulating the gut microbiota composition, particularly by reducing *Parasutterella* [81]. Moreover, dietary probiotics have been identified as a promising approach for reducing the relative abundance of the opportunistic pathogen *Parasutterella* in constipated mice [82].

The Desulfovibrionaceae family is a member of the Firmicutes phylum and is considered to be an important endotoxin producer in patients with constipation [83]. The composition of the gut microbiota in patients with slow-transmission constipation is also inconsistent with that of healthy individuals, with Desulfovibrionaceae being significantly enhanced [84]. In summary, the findings of this study demonstrate significant alterations in the gut microbiota as a whole in response to the treatment of constipation. Some microbial taxa exhibited a strong correlation with the core host parameters. However, the precise causal relationship between these changes in the gut microbiota, including some crucial microbial taxa, and the alleviation of constipation remains uncertain. To elucidate this relationship, further in-depth studies are necessary. These may include the design of fecal microbiota transplantation experiments, germ-free mice experiments, and antibiotic intervention experiments.

In addition, although the present study highlights that VID may alleviate constipation by modulating the A2AR-MLC signaling pathway, the extent to which this pathway is crucial to VID’s capacity to promote intestinal motility and alleviate constipation remains uncertain. Further comprehensive investigations are necessary to elucidate this.

## 4. Materials and Methods

### 4.1. Primary Materials

The reagents used in this experiment, including VID and phenolphthalein, were purchased from Shanghai Aladdin Biochemical Science and Technology Co. Loperamide (Shanghai, China). Hydrochloride was purchased from Sigma-Aldrich, St. Louis, MO, USA. ELISA kits for acetylcholinesterase (AChE), vasoactive intestinal peptide (VIP), and 5-hydroxytryptamine (5-HT) were purchased from Sangon Biotech (Shanghai, China) Co.

### 4.2. Design of Animal Experiments

The animal experiments complied with the ARRIVE guidelines and the UK’s Animals (Scientific Procedures) Act, 1986 and associated guidelines. All the procedures were reviewed and approved by the Life Science Ethics Committee of Yunnan Agricultural University (Approval No. 202201018). Male Kunming mice (17–22 g) were selected for the experiments and sourced from Beijing Si Pei Fu Biotechnology Co. They were fed standard chow and provided with sterilized RO water. After one week of acclimatization, the mice were randomly divided into six groups: a blank control group (CON, gavage saline twice), a model control group (LOP, 8 mg/kg loperamide and saline), a positive control group (POS, 8 mg/kg loperamide and 100 mg/kg phenolphthalein), a low-dose VID group (LVID, 8 mg/kg loperamide and 50 mg/kg VID), a medium-dose VID group (MVID, 8 mg/kg loperamide and 100 mg/kg VID), and a high-dose VID group (HVID, 8 mg/kg loperamide and 200 mg/kg VID), with 12 mice in each group, for a total of 72 mice. Phenolphthalein was used as a positive drug in this study. Loperamide and the additional drug were administered via gavage at two-hour intervals. Mice in each group were treated for seven consecutive days.

Defecation experiments and gastrointestinal transit experiments were conducted on days 7 and 8 of the subject intervention, respectively, and the specific experimental methods and steps were as in our published paper [77]. Briefly, in the defecation experiment, the first black stool time (FBST), fecal number (FN), fecal wet weight (FW), and fecal water content (FWC) of each mouse were recorded and calculated; the gastrointestinal transit ratio (GTR) of each mouse was obtained by gastrointestinal transit experiments. The blood, cecal contents, and colon tissues of the mice were collected, preserved, and kept for backup.

### 4.3. Histopathologic Analysis and Immunohistochemistry

Proximal colon samples were collected immediately after the mice were sacrificed; the fixed parts of the colon were fixed in 4% paraformaldehyde solution and then embedded in paraffin. All tissue samples were cut into 3 µm sections for hematoxylin and eosin (H&E) staining.

Immunohistochemistry was performed using previously described methods [77]. Briefly, the 3 µm sections were deparaffinized in xylene and rehydrated in a graded alcohol series. After quenching endogenous peroxidase activity and blocking non-specific binding, the slices were incubated with rabbit polyclonal to Aqp 3 (Abcam, ab125219, Shanghai, China) and Aqp 4 (Proteintech, 16473-1-AP, Wuhan, China) overnight at 4 °C, followed by incubation with a biotinylated secondary antibody (Abways, ab0101, Gainesville, FL, USA) for 30 min at room temperature. Finally, the Avidin-Biotin Complex Kit (Vector Laboratories, Burlingame, CA, USA) and the 3,3′-diaminobenzidine kit (Tiangen, Beijing, China) were used to incubate the slides. High-quality representative images of each mouse (*n* = 8) were captured with an Olympus CX43 microscope and cellSens Entry 2.2 software. The Image-Pro Plus 6.0 software (Media Cybernetics, Inc., Rockville, MD, USA) was used to digitize the resulting images.

### 4.4. RNA Extraction, Reverse Transcription, and Real-Time Quantitative PCR

First, RNA was extracted from approximately 20 mg of colon according to the instructions of the FastPure Cell/Tissue Total RNA Isolation Kit (Vazyme, Nanjing, China); then, cDNA was synthesized using the HiScript RT SuperMix for qPCR (+gDNA wiper) kit (Vazyme, Nanjing, China). Finally, the PCR system was configured using the ChamQ Universal SYBR qPCR Master Mix Kit (Vazyme, Nanjing, China), and RT-qPCR was performed on a LightCycler480 real-time fluorescence quantification system. The relative expression of target genes was calculated using the 2^−ΔΔCt^ method. The primers were selected from PrimerBank (https://pga.mgh.harvard.edu/primerbank/, accessed on 1 November 2023) and are presented in Table 1.

### 4.5. Western Blotting Analysis

Samples were lysed with RIPA lysis buffer containing the protease inhibitor PMSF (Strong, E121-01; Genstar, Shenzhen, China), and total proteins were extracted according to the manufacturer’s instructions. Protein concentrations were quantified using a BCA kit (Proteintech, PK10026; Wuhan, China). Proteins were separated by SDS-PAGE and transferred to a PVDF membrane. The membrane was then blocked with 5% bovine serum albumin for 1.5 h. Primary antibodies were polyclonal antibody against MYL9 (Proteintech, 15354-1AP; Wuhan, China), polyclonal viral antibody against β-tubulin (Proteintech, 66240-1; Wuhan, China), and all primary antibodies were incubated overnight at 4 °C. Subsequently, the corresponding secondary antibodies were incubated at room temperature for one hour at a dilution of 1:5000, followed by enhanced chemiluminescence to visualize the protein bands. ImageJ (v1.8.0) was employed to analyze the expression levels of the target proteins.

### 4.6. Serum Neurotransmitter Assay

Immediately after the mice were sacrificed, blood was collected and allowed to stand for 30 min at 37 °C, then centrifuged at 3500 rpm for 10 min at 4 °C, and serum was collected. The mouse serum was assayed for AChE and VIP according to the Elisa kit manufacturer’s instructions.

### 4.7. Analysis of Microbial Diversity of Cecum Contents

Following euthanasia, the cecum was excised, and its contents were preserved and stored in a refrigerator at -80 °C. We engaged the services of Majorbio Biotechnology Co. (Shanghai, China) to perform DNA extraction and 16S rRNA sequencing. Specifically, DNA was extracted from mouse cecal content samples using the E.Z.N.A.^®^ Soil DNA Kit (Omega Bio-tek, Norcross, GA, USA). The extracted DNA was utilized as a template for polymerase chain reaction (PCR) amplification of the V3–V4 variable region of the 16S rRNA gene of the bacterium using the forward primer 338F and the reverse primer 806R. The PCR products were purified using the PCR Clean-Up Kit (Passover, Shanghai, China) and then quantified using the Qubit 4.0 (Thermo Fisher Scientific, Waltham, MA, USA) instrument. Samples were sequenced using the Illumina PE300/PE250 platform. Sequences were processed using the QIIME 2 (v2022.2) method [85]. The majority of the data analysis was conducted on the Majorbio cloud platform (https://cloud.majorbio.com). This included α-diversity analysis, principal coordinate analysis (PCoA), species composition analysis, linear discriminant analysis effect size (LEfSe), and species difference analysis.

### 4.8. Data Analysis

The data were analyzed using GraphPad Prism 9.0 software, and the results were expressed as the mean ± standard error of the mean (Mean ± SEM). One-way ANOVA (for one variable) or two-way ANOVA (for two variables) was performed to identify significant differences among three or more groups, followed by the indicated post-hoc test (Student–Newman–Keuls comparison test), unless otherwise stated in the figure legends. The threshold for statistical significance was set to *p* < 0.05. The PCoA plot at the ASV level and genus level was based on the weighted Unifrac algorithm. The LDA score was set to >2.0 in the LEfSe analysis (phylum to genera). Bivariate correlation analysis was performed using Spearman’s method. Benjamini–Hochberg procedures (BH) were used to adjust *p*-values for multiple testing in correlation analysis. Heatmaps were generated using HemI 1.0 software (http://hemi.biocuckoo.org/down.php, accessed on 20 September 2024).

## 5. Conclusions

In this work, we systematically investigated the laxative effect of VID for the first time in FC mice. Our results indicate that VID could promote gastrointestinal peristalsis, improve fecal properties, facilitate defecation, and have a significant laxative effect on FC mice. VID was found to modulate the serum neurotransmitters, the gene expression of AQPs, and the A2AR-MLC signaling pathway in the colons of constipated mice. Additionally, VID was observed to reduce colonic inflammation, strengthen the gut barrier function, and alter the gut microbial community structure and composition. Some key microbial taxa were found to be significantly associated with important constipation phenotypes, suggesting a potential role in the alleviation of constipation by VID. These findings confirm the efficacy of VID in a constipated animal model and initially explored its molecular mechanism for relieving constipation. Further investigation is required to address several fundamental questions, including whether the A2AR-MLC signaling pathway is the primary mechanism by which VID promotes intestinal motility, and whether there is a causal relationship between VID-induced alterations in the overall gut microbiota or specific microbes and the relief of constipation. Furthermore, it would be beneficial to investigate the appropriate dosage range and safety profile of VID administration in order to establish a foundation for its prospective clinical research.

## Figures and Tables

**Table 1:** Primer sequences used for quantitative PCR analysis.

Gene	Forward Primer	Reverse Sequence	Accession Number
*IL-1β*	TCCATGAGCTTTGTACAAGGA	AGCCCATACTTTAGGAAGACA	NM_008361.4
*INF-γ*	ATCTGGAGGAACTGGCAAAA	TTCAAGACTTCAAAGAGTCTGAGGT	NM_008337.4
*IL-17*	TTTAACTCCCTTGGCGCAAAA	CTTTCCCTCCGCATTGACAC	NM_010552.3
*MCP-1*	AGCCATCCGACATTCTTC	GCCTATGCCTTCCACTTT	NM_015729.4
*AQP3*	GTCAACCCTGCCCGTGACTTTG	CGAAGACACCAGCGATGGAACC	NM_016689.2
*AQP4*	GCAGACAAGGTGCAACGTGGTT	GGCGGAAGGCAAAGCAGTATGG	NM_009700.3
*AQP8*	TGTGTAGTATGGACCTACCTGAG	ACCGATAGACATCCGATGAAGAT	NM_007474.2
*AQP9*	TGGTGTCTACCATGTTCCTCC	AACCAGAGTTGAGTCCGAGAG	NM_022026.3
*VIP*	AGTGTGCTGTTCTCTCAGTCG	GCCATTTTCTGCTAAGGGATTCT	NM_011702.3
*AChE*	CTCCCTGGTATCCCCTGCATA	GGATGCCCAGAAAAGCTGAGA	NM_001290010.1
*A2AR*	GAAGCAGATGGAGAGCCAAC	GAGAGGATGATGGCCAGGTA	NM_001428350.1
*CALM*	TGGGAATGGTTACATCAGTGC	CGCCATCAATATCTGCTTCTCT	NM_001313934.1
*smMLCK*	AGAAGTCAAGGAGGTAAAGAATGATGT	CGGGTCGCTTTTCATTGC	NM_001408263.1
*MYL9*	ACAGCGCCGAGGACTTTTC	CAGCCATTCAGCACCATCCG	BC049974
*Muc 2*	AGGGCTCGGAACTCCAGAAA	CCAGGGAATCGGTAGACATCG	NM_023566.4
*Claudin-1*	ATCACCTTCGGGAGCTCAGGT	TGATGGGGGTCAAGGGGTCAT	NM_016674.4
*Claudin-5*	TGGACCACAACATCGTGACGG	TGCCTCCCGCCCTTAGACATA	NM_013805.4
*Claudin-7*	AACATCATCACAGCCCAGGCC	ATGTTTGGAGGTGGAGTGGCC	NM_016887.6
*RPL-19*	GAAGGTCAAAGGGAATGTGTTCA	CCTTGTCTGCCTTCAGCTTGT	NM_009078.2
*β-actin*	GGCTGTATTCCCCTCCATCG	CCAGTTGGTAACAATGCCATGT	NM_007393

## Data Availability

The raw reads of 16S rRNA gene sequence data were deposited into the NCBI Sequence Read Archive (SRA) database under BioProject accession number PRJNA1125600.

## Supplementary Materials

The following supporting information can be downloaded at: https://www.mdpi.com/article/10.3390/ijms252312810/s1.

## Abbreviations

VID	Vidarabine
FC	Functional constipation
LVID	Low-VID-dose group
MVID	Medium-VID-dose group
HVID	High-VID-dose group
FBST	First black stool time
GTR	Gastrointestinal transit rate
FN	Fecal number
FW	Fecal wet weight
AChE	Acetylcholinesterase
VIP	Vasoactive intestinal polypeptide
ENS	Enteric nervous system
A2AR	Adenosine A2A receptor
smMLCK	Smooth muscle myosin light chain kinase
Calm	Calmodulin
MYL9	Myosin light chain 9
INF-γ	Interferon gamma
IL-1β	Interleukin-1β
IL-17	Interleukin-17
MCP-1	Monocyte chemoattractant protein-1
Muc2	Mucin 2
AQP3	Aquaporin 3
AQP4	Aquaporin 4
AQP8	Aquaporin 8
AQP9	Aquaporin 9
