# Supplementary material for: A Potential Use of Vidarabine: Alleviation of Functional Constipation Through Modulation of the Adenosine A2A Receptor-MLC Signaling Pathway and the Gut Microbiota
Source: Int J Mol Sci. 2024 Nov 28;25(23):12810. doi: 10.3390/ijms252312810 (PMC11641518; doi:10.3390/ijms252312810)
Supplement: Supplementary file 1 [file ijms-25-12810-s001.zip › ijms-3309420-supplementary.pdf]

A Potential Use of Vidarabine: Alleviation of Functional Constipation Through Modulation of the Adenosine A2A Receptor-MLC Signaling Pathway and the Gut Microbiota

Xiaoyu Gao [1,2,3,†], Kaifeng Guo [2,†], Shuangfeng Liu [2], Weixing Yang [2], Jun Sheng [3], Yang Tian [1,3], Lei Peng [2,*] and Yan Zhao [4,*]

[1] Yunnan Key Laboratory of Precision Nutrition and Personalized Food Manufacturing, Yunnan Agricultural University, Kunming 650201, China; 2018014@ynau.edu.cn (X.G.); tianyang@ynau.edu.cn (Y.T.)
[2] College of Food Science and Technology, Yunnan Agricultural University, Kunming 650201, China; guo0446@163.com (K.G.); 2021210106@stu.ynau.edu.cn (S.L.); 2021110006@stu.ynau.edu.cn (W.Y.)
[3] Engineering Research Center of Development and Utilization of Food and Drug Homologous Resources, Ministry of Education, Yunnan Agricultural University, Kunming 650201, China; shengj@ynau.edu.cn
[4] Division of Science and Technology, Yunnan Agricultural University, Kunming 650201, China
* Correspondence: 2015042@ynau.edu.cn (L.P.); 2021013@ynau.edu.cn (Y.Z.)
† These authors contributed equally to this work.

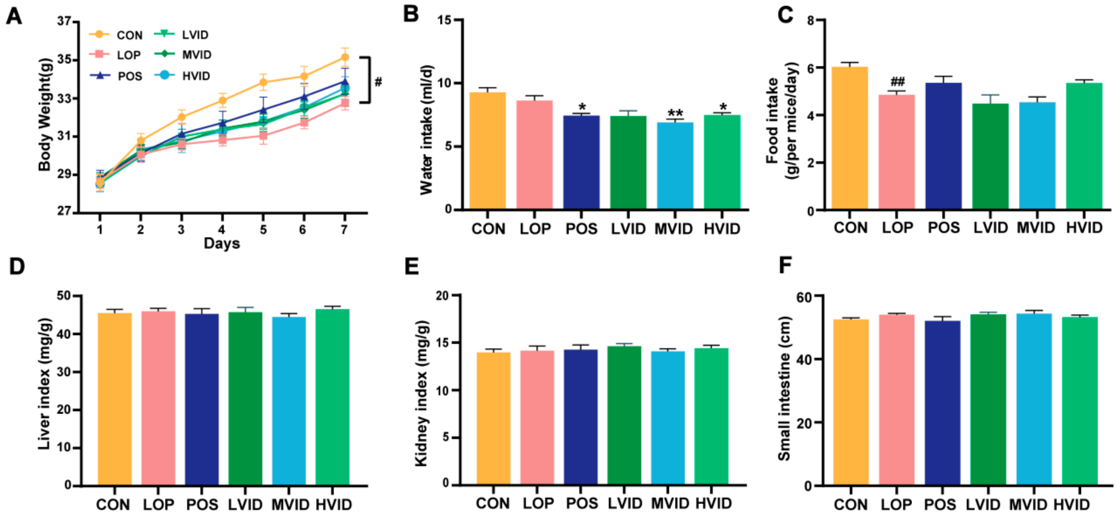

Figure S1. Effects of VID on loperamide-induced constipation symptoms in mice. (A)Body weight; (B)Water intake; (C)Food intake; (D)Liver index; (E)Kidney index; (F)Length of small intestine.

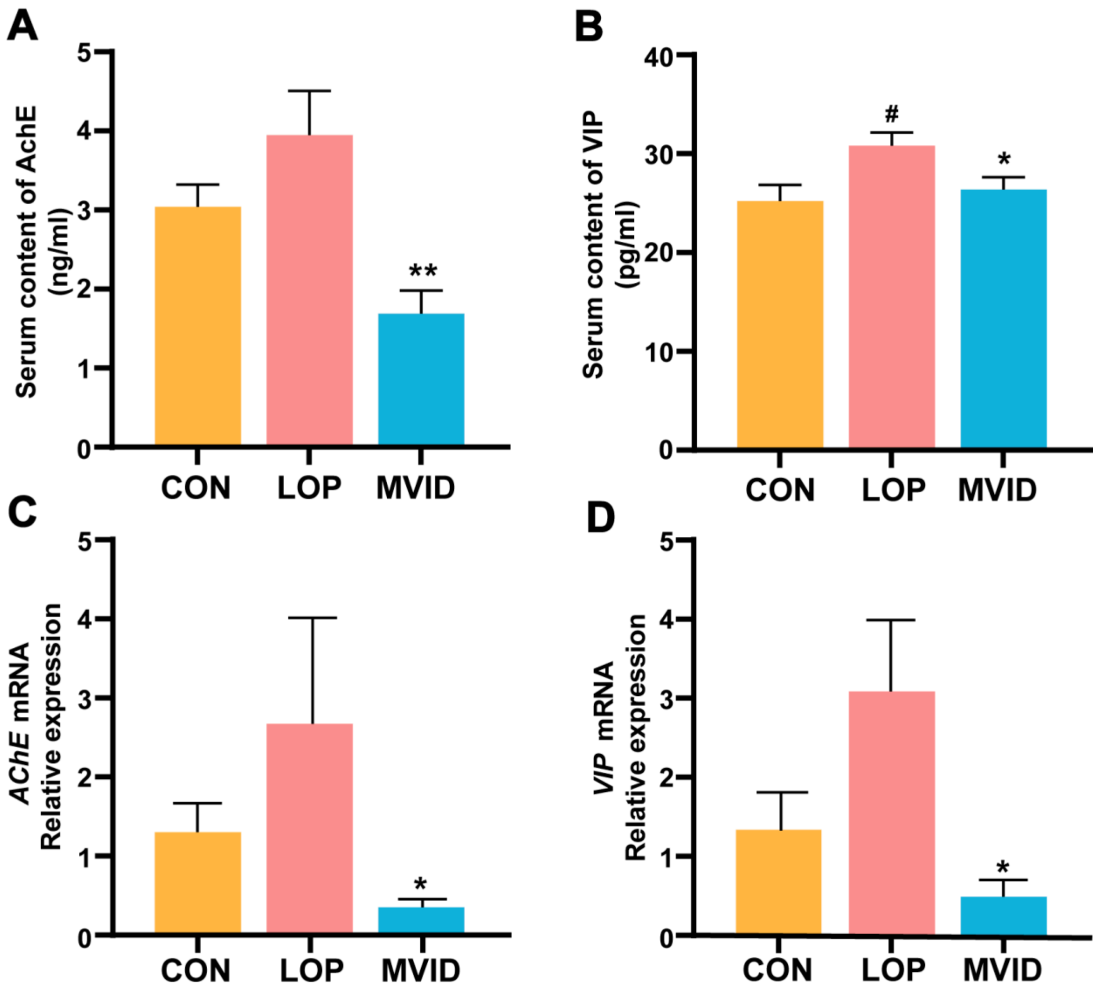

Figure S2 Effect of Vidarabine on neurotransmitter related factors in mice

A. Serum acetylcholinesterase (AChE) levels; B. Serum vasoactive intestinal peptide (VIP) levels; C. The relative mRNA expression of *AChE* in the colon; D. The relative mRNA expression of *VIP* in the colon; The data are presented as the mean ± SEM (n = 6). #, vs. CON group; *, vs. LOP group. #, $P < 0.05$. *, $P < 0.05$; **, $P < 0.01$.

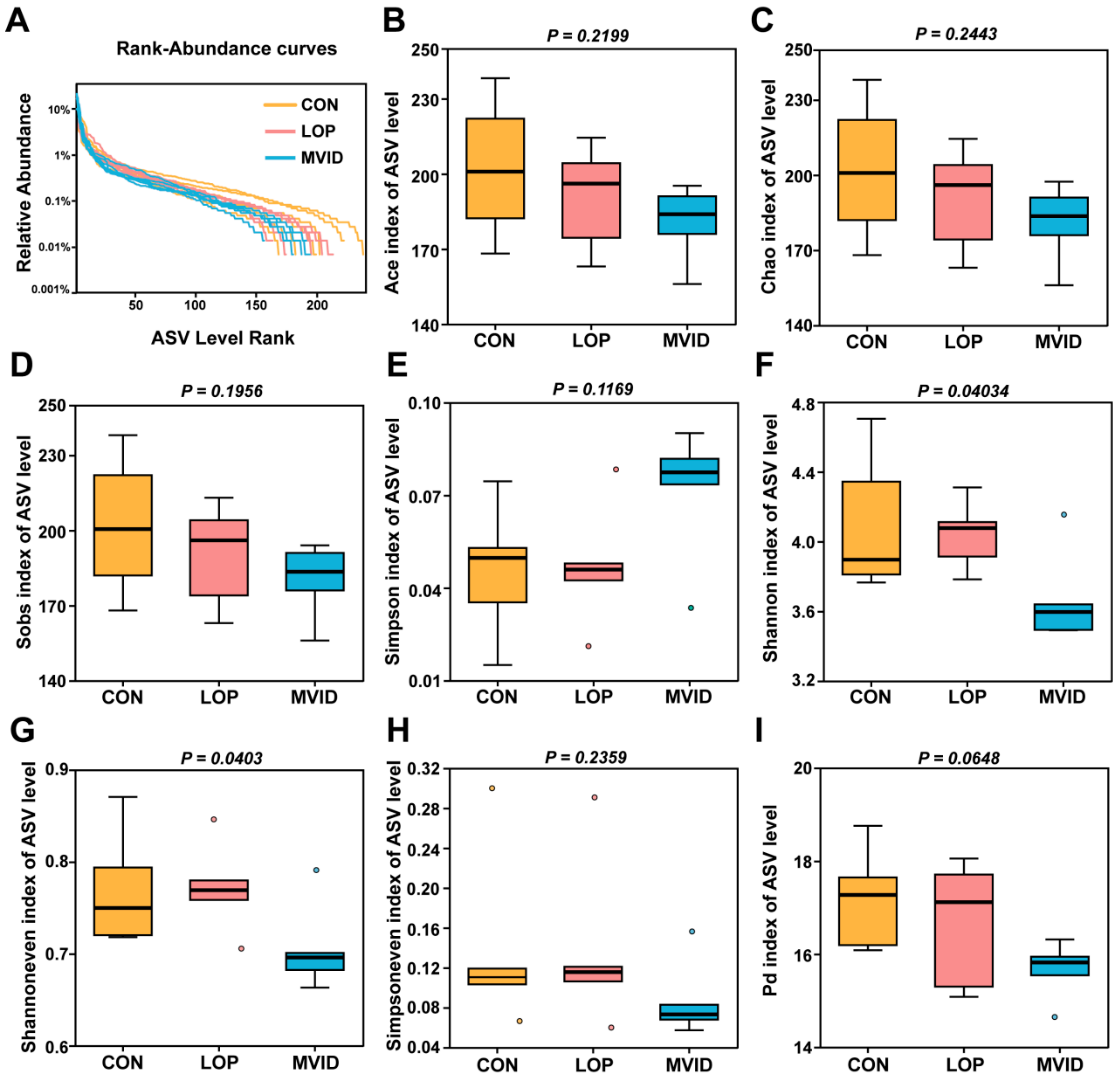

Figure S3 Effect of VID on the alpha diversity of the cecal microbiota in constipated mice. (A) Rank-Abundance curve. (B-I) Ace, Chao, Sobs, Simpson, Shannon, Shannoneven, Simpsoneven, and Pd.

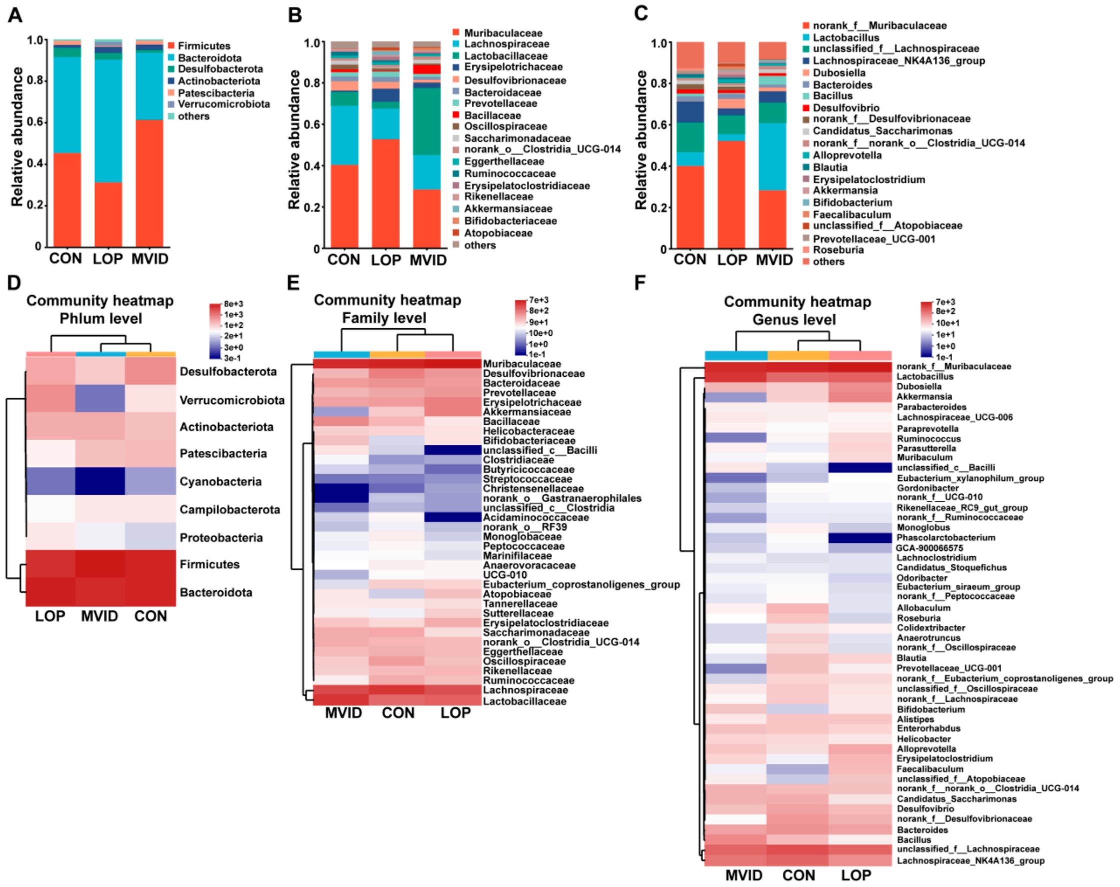

Figure S4 Effect of VID on the cecum microbial composition of mice. (A) Phylum level. (B) Family level. (C) Genus level. (D to F) Cluster heatmaps of gut microbiota in different groups.

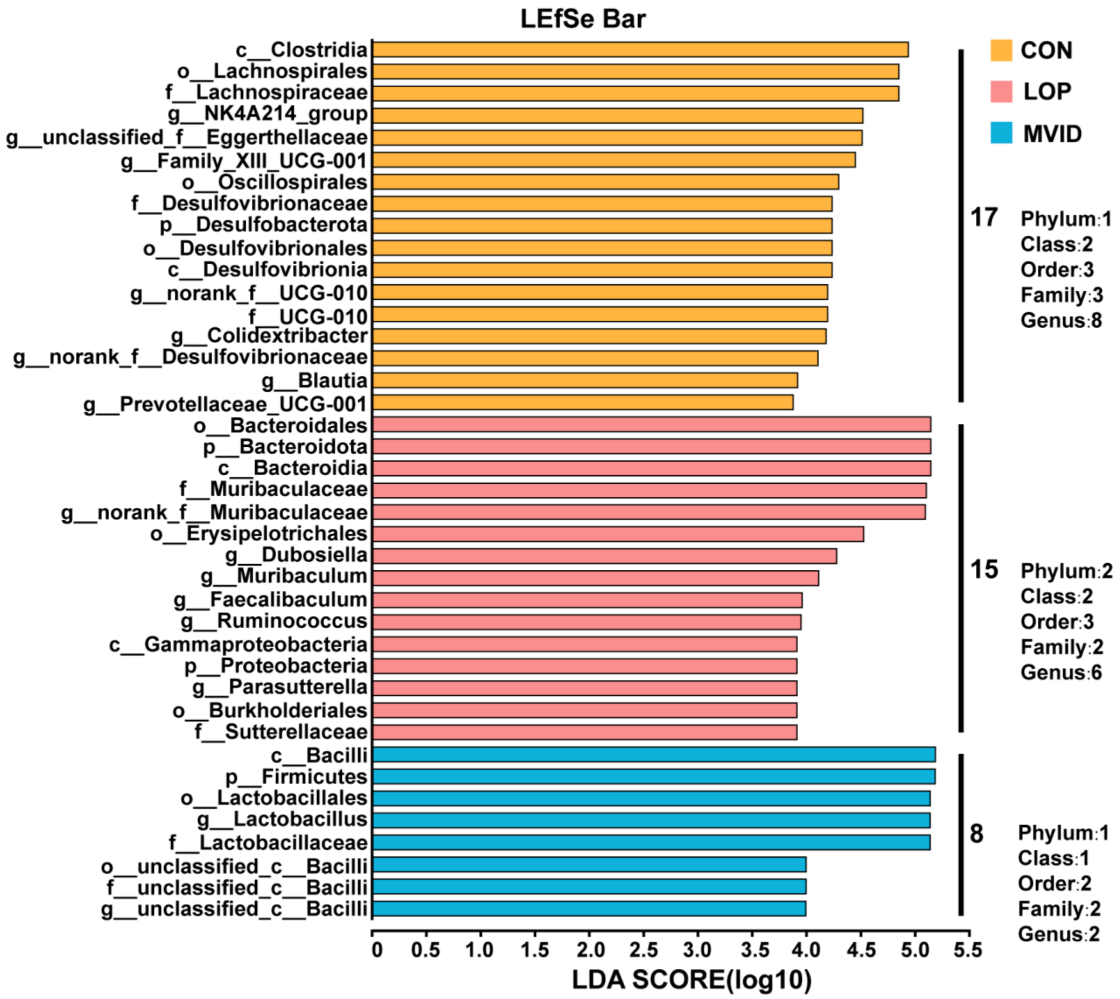

Figure S5 LEfSe analysis based on the CON, LOP and HCC groups (LDA score > 2.0).
